# Editorial: Genetic and Epigenetic Insights Into the Developmental Origins of Health and Disease

**DOI:** 10.3389/fgene.2021.814126

**Published:** 2022-02-04

**Authors:** Daniel A. Enquobahrie, Fasil Tekola-Ayele, Tesfaye B. Mersha

**Affiliations:** ^1^ Department of Epidemiology, School of Public Health, University of Washington, Seattle, WA, United States; ^2^ Epidemiology Branch, Division of Population Health Research, Division of Intramural Research, Eunice Kennedy Shriver National Institute of Child Health and Human Development, National Institutes of Health, Bethesda, MD, United States; ^3^ Cincinnati Children’s Hospital Medical Center, Department of Pediatrics, University of Cincinnati College of Medicine, Cincinnati, OH, United States

**Keywords:** genetics, epigenetics, environmental determinants, pre-natal, adulthood, childhood

The developmental origins of health and disease hypothesis posits that perturbations in the *in-utero* environment contribute to functional and metabolic programming as well as structural adaptations of fetal tissues predisposing the individual to chronic diseases during childhood and adulthood (Hales et al., 1991). Genetic and epigenetic studies document risk factors and molecular mechanisms that contribute to shared pathogenetic pathways between early life outcomes such as fetal growth and later-life chronic diseases. These studies have shown that shared genetic effects, non-genetic factors (such as social and environmental factors), as well as developmental programming can explain the relationships between early life and later life outcomes (Warrington et al., 2019; Tekola-Ayele et al., 2020a; Tekola-Ayele et al., 2020b; Juliusdottir et al., 2021).

Integrated genetic and epigenetic studies involving pregnant women, the placenta, and the offspring can lead to novel discoveries of molecular signals of early origins of childhood and adulthood diseases. The prevalence and disparity of complex diseases (across racial/ethnic groups) with early life origins has been increasing in the United States and around the world in recent decades. However, few studies integrate genetic, epigenetic, social, and environmental determinants of early life phenotypes to understand their links with diseases in later life. In this *Research Topic*, we gathered articles on genetic and epigenetic factors and their influences on pregnancy outcomes, and childhood and adult diseases.

Mancilla et al. reviewed the literature to examine health inequality within the context of social epigenomics. Sasaki et al. investigated the effect of sample handling on DNA methylation profiles. Candelo et al. investigated a possible association between Zika virus infection and cyclin-dependent kinase 5 regulatory subunit-associated protein 2 (CDK5RAP2) mutation. Le et al. investigated the mechanisms linking assisted reproductive technology (ART) to cholesterol metabolic and respiratory disorders later in life. Xu et al. investigated the use of maternal serum human leukocyte antigen-G (sHLA-G) to detect prenatal chromosomal abnormalities. Ferreira and Dantas Junior reported a case study of a neonate with Beare-Stevenson Syndrome whose father had Congenital Bilateral Absence of the Vas Deferens (CBAVD). Luo et al. presented a whole exome sequencing study of Joubert Syndrome (JBTS), a type of ciliopathies.

This topical collection presents original research, review articles, and case studies on a scope of exposures and health outcomes spanning the pre-natal period through adulthood. Future studies integrating a spectrum of genetic and epigenetic studies along with relevant exposures (including environmental exposures and lifestyle) have a potential to inform mechanisms that underlie the associations between maternal phenotypes, birth outcomes, and offspring adult diseases.
